# Quantitative 3-T multi-parametric MRI and step-section pathology of recurrent prostate cancer patients after radiation therapy

**DOI:** 10.1007/s00330-018-5819-y

**Published:** 2018-11-12

**Authors:** Catarina Dinis Fernandes, Ghazaleh Ghobadi, Henk G. van der Poel, Jeroen de Jong, Stijn W. T. P. J. Heijmink, Ivo Schoots, Iris Walraven, Petra J. van Houdt, Milena Smolic, Floris J. Pos, Uulke A. van der Heide

**Affiliations:** 1 Department of Radiation Oncology, The Netherlands Cancer Institute, Plesmanlaan 121, 1066 CX Amsterdam, The Netherlands; 2 Department of Urology, The Netherlands Cancer Institute, Amsterdam, The Netherlands; 3 Department of Pathology, The Netherlands Cancer Institute, Amsterdam, The Netherlands; 4 Department of Radiology, The Netherlands Cancer Institute, Amsterdam, The Netherlands

**Keywords:** Prostate Cancer, Local neoplasm recurrence, Magnetic resonance imaging, Pathology, Radiotherapy

## Abstract

**Objectives:**

Diagnosis of radio-recurrent prostate cancer using multi-parametric MRI (mp-MRI) can be challenging due to the presence of radiation effects. We aim to characterize imaging of prostate tissue after radiation therapy (RT), using histopathology as ground truth, and to investigate the visibility of tumor lesions on mp-MRI.

**Methods:**

Tumor delineated histopathology slides from salvage radical prostatectomy patients, primarily treated with RT, were registered to MRI. Median T2-weighted, ADC, *K*^trans^, and *k*_ep_ values in tumor and other regions were calculated. Two radiologists independently performed mp-MRI-based tumor delineations which were compared with the true pathological extent. General linear mixed-effect modeling was used to establish the contribution of each imaging modality and combinations thereof in distinguishing tumor and benign voxels.

**Results:**

Nineteen of the 21 included patients had tumor in the available histopathology slides. Recurrence was predominantly multifocal with large tumor foci seen after external beam radiotherapy, whereas these were small and sparse after low-dose-rate brachytherapy. MRI-based delineations missed small foci and slightly underestimated tumor extent. The combination of T2-weighted, ADC, *K*^trans^, and *k*_ep_ had the best performance in distinguishing tumor and benign voxels.

**Conclusions:**

Using high-resolution histopathology delineations, the real tumor extent and size were found to be underestimated on MRI. mp-MRI obtained the best performance in identifying tumor voxels. Appropriate margins around the visible tumor-suspected region should be included when designing focal salvage strategies. Recurrent tumor delineation guidelines are warranted.

**Key Points:**

*• Compared to the use of individual sequences, multi-parametric MRI obtained the best performance in distinguishing recurrent tumor from benign voxels.*

*• Delineations based on mp-MRI miss smaller foci and slightly underestimate tumor volume of local recurrent prostate cancer.*

*• Focal salvage strategies should include appropriate margins around the visible tumor.*

**Electronic supplementary material:**

The online version of this article (10.1007/s00330-018-5819-y) contains supplementary material, which is available to authorized users.

## Introduction

Depending on the risk group, 5-year disease-free survival varies from 67 to 80% for prostate cancer patients who are treated with whole-gland radiation therapy (RT) to 78 Gy [1]. Most patients with recurrent disease have developed metastasis, yet a small but significant proportion will harbor locally recurrent disease only [2, 3]. For these patients, salvage treatment options include radical prostatectomy, brachytherapy, cryotherapy, or high-intensity focused ultrasound [4]. Whole-gland approaches, such as salvage radical prostatectomy (SRP), are reported to obtain good disease control, however with a high chance of severe gastrointestinal and genitourinary complications when compared with radical prostatectomy in primary disease [5].

Focal salvage strategies aim at reducing these comorbidities by sparing the uninvolved tissue while specifically targeting recurrent cancer. When evaluated in radio-recurrent PCa, the complications of focal approaches are comparable or lower than in whole-gland strategies, with a 5-year biochemical disease-free survival of 46.5–54.5% [4].

Successful development of focal salvage strategies for local recurrent PCa requires an accurate detection and localization of the tumor. Positron emission tomography (PET) with ^68^Ga PSMA ligands offers high sensitivity to detect recurrent PCa [6], yet the poor spatial resolution limits its use for focal salvage strategies. Magnetic resonance imaging (MRI) alongside PET is the most used modality for recurrence diagnosis. High-resolution anatomical and functional imaging makes MRI attractive for the preparation of focal salvage treatments [7].

Post-RT benign confounders pose a challenge to MRI interpretation. Prostate tissue shows diffuse signal intensity (SI) reduction on T2-weighted (T2w) MRI, complicating tumor detection [8]. The use of 1.5-T MR spectroscopy [9] as well as DWI- and DCE-MRI [10] has been found to surpass T2w in detecting local recurrence. Thus, multi-parametric MRI (mp-MRI) with diffusion-weighted imaging (DWI) and dynamic contrast-enhanced (DCE) MRI is often preferred. Zattoni et al [11] reported 3-T mp-MRI to have good accuracy in detecting recurrent disease extension. Conversely, Donati et al [12] found no additional benefit of DCE when added to T2w and DWI. These studies used either biopsy or SRP samples for validation, but none attempted accurate registration of imaging and histopathology. To date, no guidelines exist on how to score or delineate recurrent tumor-suspected regions.

In this study, 3-T mp-MRI is used to characterize irradiated prostate tissue, using SRP specimens registered to mp-MRI as ground truth. Using high-resolution tumor delineations in histopathology, we describe radio-recurrent PCa and use mixed modeling to establish which imaging sequences result in an optimal distinction between tumor and benign voxels. We further investigate the accuracy of tumor detection in mp-MRI, a crucial element for the development of focal salvage strategies.

## Materials and methods

### Patients

Twenty-one patients with radio-recurrent PCa, who underwent mp-MRI for local staging prior to SRP between 2011 and 2017, were retrospectively included. Biochemical recurrence was established according to the Phoenix criteria [13]. All patients had biopsy-proved recurrence and absence of distant metastasis on choline PET scans. External beam radiotherapy (EBRT)- and low-dose rate (LDR) brachytherapy-treated patients were included.

### MRI acquisition

Patients were scanned on a 3T Achieva (16), Achieva dStream (3), or Ingenia (2) MRI scanner (Philips Healthcare) between June 2011 and March 2017. Thirteen patients were scanned using an endorectal coil in addition to a torso or cardiac phased array coil. The mp-MRI protocol included triplanar T2w turbo spin-echo (TE 90–130 ms, TR > 2600 ms) and axial T1-weighted (T1w) gradient echo sequences (TE < 2.3 ms, TR < 5.3 ms). DWI was acquired using a single-shot spin-echo echo-planar imaging sequence (*b*-values between 100 and 1000s/mm^2^), from which apparent diffusion coefficient (ADC) maps were generated. DCE was acquired with a 3D T1w spoiled gradient echo sequence (TE/TR 2/4 ms), dynamic interval 2.3–2.9 s over 5–6 min, with intravenous administration of 7.5 mmol gadoteric acid (Dotarem). For seven patients, the DCE sequence was not acquired. The pharmacokinetic parameter maps *K*^trans^ and *k*_ep_ were derived from the DCE scans. A balanced steady-state free precession (bSSFP) sequence was acquired for fiducial and seed visualization in seven patients. MRI acquisition and post-processing details can be found in the Supplementary materials.

Visual inspection was used to assess displacements between the functional sequences and the T2w scans, with rigid registration performed when necessary. The T2w voxel size in plane resolution was between 0.3 and 0.8 mm, with a slice thickness of 3 mm. All maps were resampled to the T2w grid.

### Pathology

Whole-mount axial slides, stained with hematoxylin and eosin (H&E), were used for histopathological validation. Slides at the apex and base of the specimen were sliced parasagitally for extra-prostatic extension evaluation and were excluded from analysis. The slides were digitalized using an Aperio ScanScope XT (Aperio Technologies). Supervised by an uro-pathologist (6 years of experience), tumor delineations were made (resolution 0.5 μm/pixel) on the digitized slides using ImageScope.

### T2w MRI and H&E registration

The registration between H&E slides and the transversal T2w MRI was performed by two observers in consensus. A T2w slice was visually assigned to each H&E slide. Slide matching was performed considering the order of the slides, the location of the apex and base of the prostate, visible anatomical landmarks, and the relative size and shape of subsequent H&E and T2w slices. Each H&E slide was then registered to its matched T2w slice using deformable registration based on landmark points (Coherent Point Drift) [14] implemented in MATLAB R2015a (The MathWorks). Landmark points included the urethra, nodules, the prostate boundary, and the tumor.

The registration error was estimated by selecting one landmark per pathology slide and measuring the Euclidean distance between the point in the T2w and in the registered pathology.

After registration, tumor delineations on the H&E slides were propagated to MRI. To ensure that only tumor voxels were used to characterize imaging and train the mixed model, tumor delineations were eroded in all directions by 1 mm.

All further analyses were restricted to the MRI slices for which a matching H&E slide existed.

### Region of interest segmentation

A ROI was delineated in the levator ani muscle and the median SI in this region was used to normalize the T2w images. For MRI slices with H&E match, the entire prostate and peripheral zone (PZ) were delineated using the transversal T2w MRI. The central gland (CG) was defined as the remaining non-PZ region, comprising the central and transition zone. The periurethral (PU) region was delineated with both sagittal and transversal T2w MRI. All voxels within these regions were included in the analysis. The location of implanted seeds and fiducial markers, as visible in the T1w or bSSFP sequences, was removed from the analyses.

Two uro-radiologists (14 and 7 years of experience) independently delineated suspected tumor regions, having access to mp-MRI and the radiological report, the PET choline scans, and the biopsy results. Since PI-RADS v2 [15] is not applicable to recurrent prostate cancer, tumor was defined as a region with low signal intensity (SI) on T2w MRI, high SI in the *b* = 800 DWI scan, low SI on the ADC map, and increased enhancement in the *K*^trans^ and *k*_ep_ maps. Tumor regions delineated on mp-MRI were compared with the delineations propagated from histopathology, and the distance and overlap were described. When overlapping, the distance and spatial overlap were quantified using the 95% Hausdorff distance between contours and the Dice coefficient. Delineated and pathological volumes were estimated using the T2w grid.

For LDR brachytherapy patients with seminal vesicle (SV) invasion, the visible tumor areas as well as a benign region were delineated but no distances with histopathology contours were determined.

### Statistics

Significant differences (*p* < 0.05) of median imaging values between ROIs were tested with a non-parametric Friedman’s ANOVA. To further examine differences, a post hoc Wilcoxon signed rank test was used with a Bonferroni correction.

Using the EBRT patients, univariate and multivariate generalized linear mixed-effect modeling was applied to assess the predictive value of imaging on the voxel-wise likelihood of tumor. Voxels were grouped as follows: benign, resulting from a combination of unaffected PZ and CG for which H&E was available, and tumor, based on pathology delineations. To obtain the likelihood of tumor on a voxel level, fixed and random effects were included.

The T2w, ADC, *K*^trans^, *k*_ep_ maps, and voxel anatomical location (PZ or CG) were included as fixed effects. Random effects accounted for spatial clustering, by incorporating the voxel relative distance from the prostate center of mass and patient identifiers. When the association between model parameters and tumor probability was nonlinear, the parameters were grouped in quartiles and regression coefficients were estimated for each group considering the first quartile as reference. The model fit was assessed using the Bayesian Information Criterion (BIC) (a decrease of 10 points reflects an improved fit) and by evaluating the residual random error. The contribution of predictors such as time since RT, use of hormonal therapy (HT), and treatment modality, included as fixed effects, was also investigated. A calibration curve was used to assess the quality of the best model. Analyses were performed in R (RStudio) using the lme4 package.

## Results

Thirteen patients had been primarily treated with EBRT and eight with LDR brachytherapy between 1999 and 2013. Median time between treatment and the MRI for recurrence diagnosis was 84 months for EBRT and 60 months for LDR brachytherapy. Following the D’Amico definition [16], 1, 8, and 12 patients had low-, intermediate-, and high-risk primary PCa. Eleven patients received HT as part of their primary treatment, but none received HT at the time of imaging. Patient characteristics can be found in Table 1. Gleason score was not assigned to the recurrent tumor as radiation-induced atypia can be a confounder for pathological interpretation [17].

**Table 1:** Patient characteristics

Nadir PSA (ng/ml) [IQR]	0.7 [0.9]
Median PSA level at MR imaging (ng/ml) [IQR]	5.4 [3.4]
Median time from MRI to SRP (months) [IQR]	3 [3]
Median iPSA level (ng/ml) [IQR]	15 [22]
Primary clinical tumor stage	
T1c	4
T2a	4
T2b	2
T2c	1
T3a	7
T3b	3
Primary Gleason grade	
Gleason 6	6
Gleason 7	
7 (3 + 4)	10
7 (4 + 3)	3
Gleason 8 (5 + 3)	1
Gleason 9 (5 + 4)	1
Recurrent pathological tumor stage	
pT2b	1
pT2c	4
pT3a	3
pT3b	9
pT3c	3
pT4a	1

*IQR* interquartile range

### Pathological findings

EBRT patients had an average tumor volume of 1.2 cm^3^, LDR patients 0.51 cm^3^. For all patients except one, recurrent tumor after LDR brachytherapy extended to the SV. In this subset, tumor within the gland was mainly small and sparse. For 2 patients, no tumor was visible in the available H&E slides, with their pathology reports describing tumors of small dimensions in the prostate apex, bladder neck, and/or extension to the SV. These patients were excluded from further analysis.

**Fig. 1:**
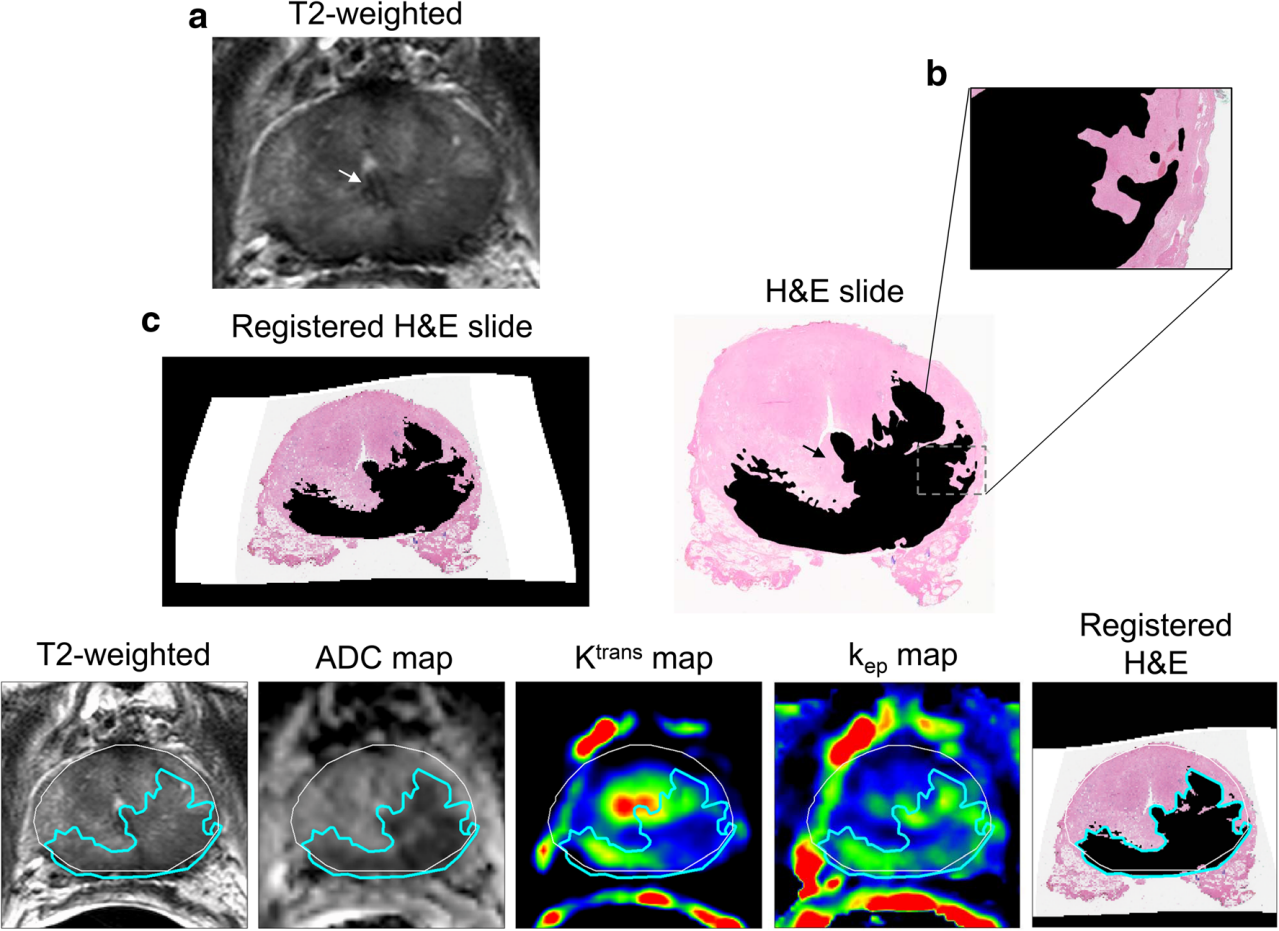
Example of an EBRT patient. The white arrow points at the urethra, location used for the match of the H&E slide (**b**) with T2w MRI (**a**). The registered H&E slide (**c**) is placed side by side with the mp-MRI images where the prostate is delineated in white and the tumor in blue

The average registration error for all slides was 1.1 ± 0.9 mm. Quantitative image analysis based on the matched H&E slides was performed for 19 patients and 56 slides (Fig. 1). Multifocal recurrent disease was present for all but one patient. Sixty-three percent of the tumors were located in the PZ and 11% in the CG. The remaining 26% covered both PZ and CG. Benign prostatic hyperplasia (BPH) could not be seen in the pathology slides matched to MRI. The pre-SRP biopsy report contained information about the location of positive cores for 17/21 patients. When compared to the SRP histopathology, biopsy correctly detected 42% of SV-located tumors, as well as 33% of left and 75% of right prostate gland tumors.

### Radiologists’ delineations compared to histopathology

Median delineated volumes (cm^3^) were 0.34 (range, 0.04–2.14) for radiologist 1 and 0.56 (range, 0.06–3.50) for radiologist 2, and missed tumor foci were smaller than 1 and 0.5 cm^3^, respectively.

**Fig. 2:**
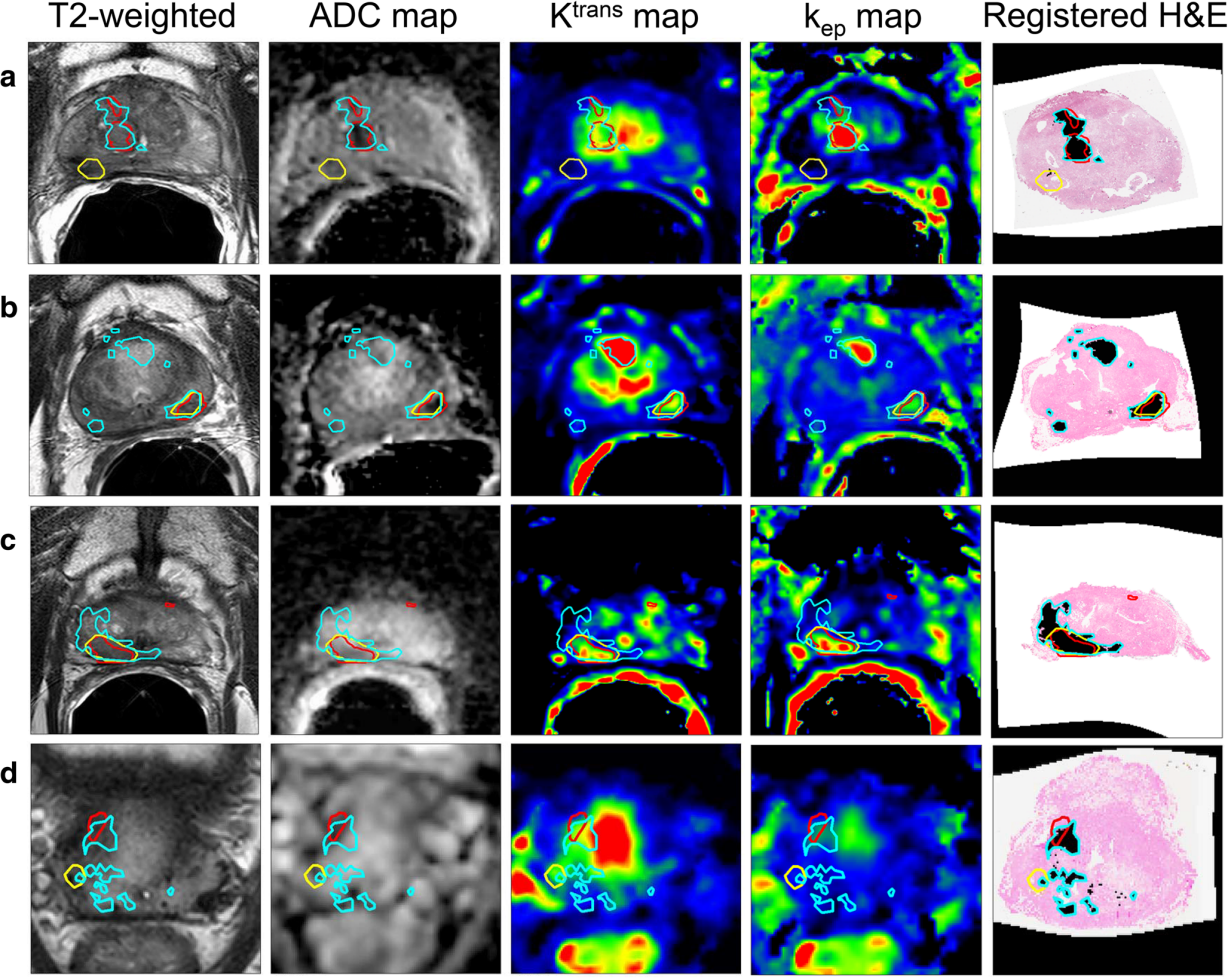
Example patients treated with primarily EBRT (**a-c**) and LDR brachytherapy (**d**) with histopathology delineations propagated to MRI (in blue) and tumor-suspected regions delineated by the experienced uro-radiologists (in yellow and red)

Radiologists’ contours did not visually overlap with pathology for 5 patients, with 3 of them missed by both. When overlapping with pathology, the median delineated volumes (cm^3^)—0.61 (range, 0.05–1.76) and 0.68 (range, 0.06–2.82)—slightly underestimated the corresponding median pathological tumor volumes (cm^3^)—0.74 (range, 0.01–2.82) and 0.74 (range, 0.02–2.82) (Fig. 2(A–C)). Contours overlapping with pathology had a median Dice coefficient of 0.64 (range, 0.1–0.8) and 0.58 (range, 0.01–0.83) and a median 95% Hausdorff distance (mm) of 3.6 (range, 0.4–20.9) and 6.2 (range, 0.9–17.1) for radiologists 1 and 2 respectively.

The pattern of recurrence after LDR brachytherapy, showing small and sparse foci (Fig. 2(D)), together with the artifacts caused by the seeds, made detection of recurrent tumor within the gland challenging. Disease extension to the SV was more readily visible as it presented characteristic tumor features on MRI.

### MR imaging parameters

**Fig. 3:**
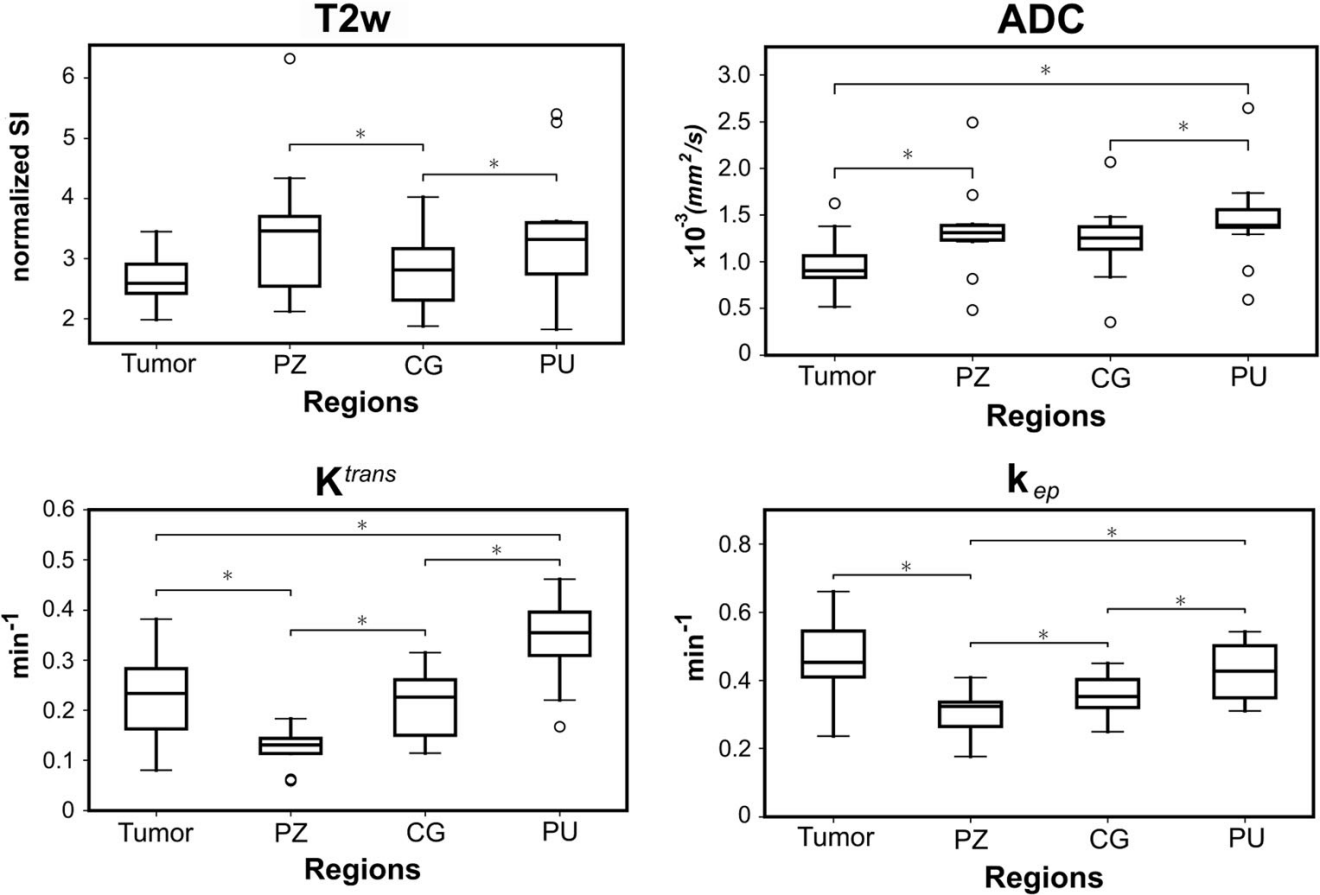
Median imaging values for all ROIs of the 13 EBRT patients. The boxes represent the first (25th) and third (75th) quartile; the horizontal line indicates the median and the whiskers the limit *Q*_1_ − 1.5 × *Q*_1_ and *Q*_3_ + 1.5 × *Q*_3_; dots represent outliers. The asterisk denotes significant differences (*p* < 0.008)

Diffuse SI reduction throughout the prostate and a decreased conspicuity of zonal anatomy was observed on T2w. For most patients (10/17 with DCE), the PU region showed *K*^trans^ enhancement without signs of malignancy (Fig. 1). Quantitative imaging values are reported for all ROIs in Fig. 3 for EBRT and Supplementary Fig. 1 for LDR brachytherapy patients. Further information about the values per ROI can be found in Supplementary Table 1.

**Fig. 4:**
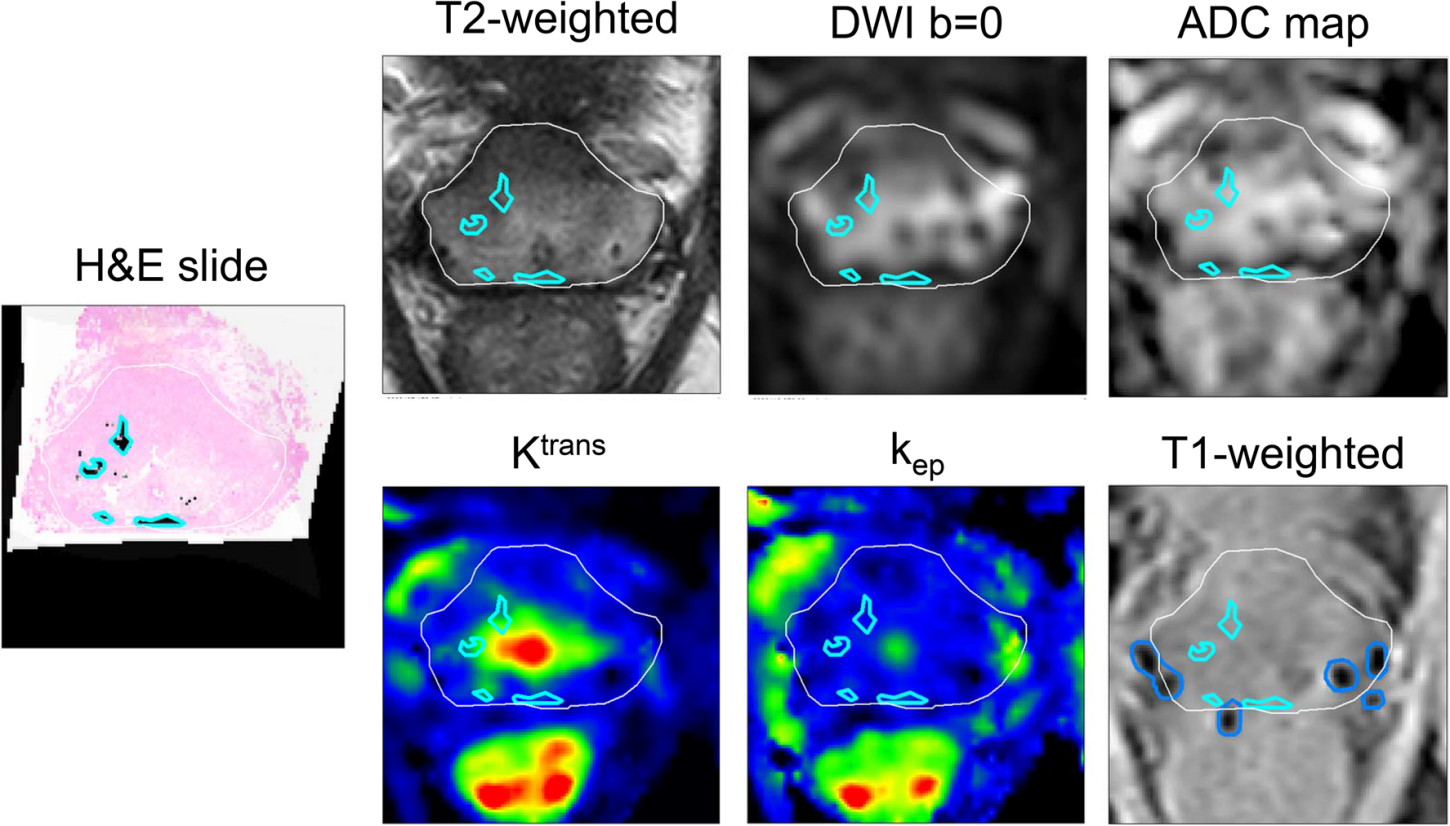
Example of a LDR brachytherapy patient in which the DWI sequence is distorted by the presence of the seeds. The region masked out of the analysis due to the presence of the seeds (in dark blue) was delineated using the T1w gradient echo sequence

#### EBRT patients

Friedman’s ANOVA test revealed significant differences between regions. A Wilcoxon signed rank test between tumor and PZ, with a corrected *α* = 0.05/6 = 0.008, found median ADC (PZ = 1.3 × 10^−3^ mm^2^/s, tumor = 0.9 × 10^−3^ mm^2^/s), *K*^trans^ (PZ = 0.13 min^−1^, tumor = 0.23 min^−1^), and *k*_ep_ (PZ = 0.32 min^−1^, tumor = 0.45 min^−1^) to be significantly different. No imaging modality was significantly different between tumor and the CG. The PU region had the highest values for all but *k*_ep_, with *K*^trans^ (PU = 0.35 min^−1^) and ADC (PU = 1.4 × 10^−3^ mm^2^/s) significantly higher than tumor. For T2w and *K*^trans^, there was a considerable overlap between tumor and CG values (Fig. 3). The tumor had the lowest T2w and ADC and the highest *k*_ep_ values.

#### LDR brachytherapy patients

For LDR brachytherapy patients, no significant differences were found between ROIs for all imaging modalities (Supplementary Fig. 1). The majority of the foci in the gland were small and did not have typical tumor characteristics on mp-MRI. The DWI was degraded due to the presence of the seeds (Fig. 4**)**. Similarly to EBRT patients, the PU had the highest values for all but *k*_ep_ maps. For the seven patients with SV invasion (Table 2**)**, tumor in the SV had the highest values for *K*^trans^ and *k*_ep_ and lowest ADC and T2w values.

**Table 2:** Median imaging values for tumor and benign ROIs in the seminal vesicles for the LDR brachytherapy patients presented as median (Q10–Q90)

Imaging parameter	Seminal vesicles	
	Tumor	Benign
T2w		
Normalized T2 values (SI)		
Median	2.9 (2.4–4.1)	6.6 (4.2–8.4)
DWI		
ADC (× 10^−3^ mm^2^/s)		
Median	1.0 (0.9–1.9)	1.7 (1.3–3.2)
DCE		
*K*^trans^ (min^−1^)		
Median	0.20 (0.17–0.33)	0.13 (0.06–0.22)
*k*_ep_ (min^−1^)		
Median	0.59 (0.48–0.79)	0.47 (0.29–0.55)

### Mixed modeling

**Fig. 5:**
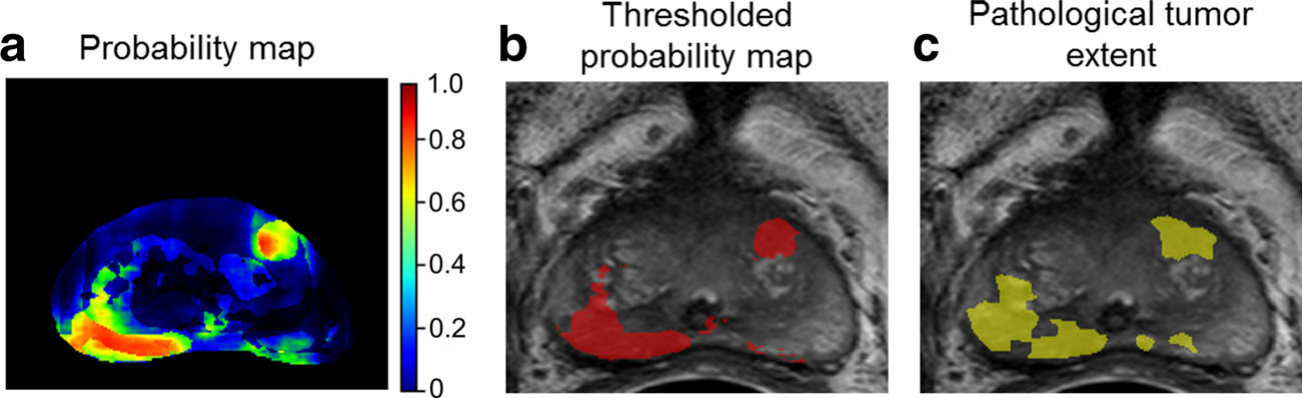
**a** Tumor likelihood (with probabilities between 0 and 1) for every prostate voxel, obtained with the use of the multivariate mp-MRI model. **b** Region with probability values above 0.5 (red overlay on the T2w). **c** True pathological extent based on the matched H&E slide (yellow overlay on the T2w)

Univariately, all MRI parameters were significantly associated with tumor likelihood, *k*_ep_ having the best statistical performance (BIC of 239,552, compared to 286,265, 247,260 and 254,273 for T2, ADC, and *K*^trans^). Location (PZ = 0 or CG = 1) was the only non-imaging parameter that improved discrimination, with a voxel location in the CG decreasing tumor likelihood. A multivariate model combining all mp-MRI parameters and location obtained the best fit and was the most predictive for tumor (BIC of 198,942, *p* < 0.001) (Supplementary Table 2). A calibration curve for this model can be found in (Supplementary Fig. 2), showing that model predicted probabilities correspond well to the actual fractions of voxels with tumor in histopathology. Figure 5 shows an example of a reconstructed tumor probability map originating from the use of the mp-MRI model.

## Discussion

In selected patients, focal salvage can achieve local control while reducing the risk of adverse events. An accurate representation of tumor location is necessary and mp-MRI has been used for this purpose [18–22]. In a quadrant analysis, T2w alone was reported to result in a similar tumor evaluation accuracy as in the pretreatment setting, but with substantial interobserver variability [23]. Benign confounders in previously irradiated prostates can complicate tumor detection. No consensus exists yet on the optimal MRI sequences for detecting local recurrence. Since patients only sporadically receive SRP after RT, and with limited availability of mp-MRI prior to surgery, studies reporting on these patients are usually based on small numbers [9, 23, 24]. Our study is unique in registering pathology to MRI to propagate high-resolution tumor delineations.

Consistent with literature, we found radio-recurrence disease to be often multifocal [25] yet with a different recurrence pattern in EBRT- and LDR brachytherapy-treated patients. No BPH was found in the available pathological slides, suggesting that it is not an imaging confounder as in the untreated prostate.

Our findings have implications for the design of focal salvage treatments. Smaller foci were often missed, and when overlapping with pathology, radiologists’ delineations often underestimated the real tumor size. Both radiologists performed quite similarly regarding the evaluated metrics. Our results are in line with findings in the de novo setting, comparing mp-MRI tumor detection to prostatectomy specimens: Borofsky et al [26] reported that in 26% of the patients, clinically important lesions were missed and in 8% there was substantial tumor size underestimation; according to Bratan et al [27], two observers underestimated tumor volume with every pulse sequence; and Steenbergen et al [28] described how teams of observers missed all small satellites and parts of the 18/22 correctly detected dominant lesions. In the setting of recurrent PCa, tumor volume underestimation in 1.5-T T2w MRI has been described using SRP samples [24]. The 95% Hausdorff distances reported here were above the 2.3 mm error observed in primary tumor delineations on mp-MRI [28]. Our results suggest a margin could improve index lesion coverage in focal salvage strategies. The development of MRI scoring and recurrent tumor delineation guidelines would likely improve consistency between observers and studies.

Analysis of the quantitative imaging maps showed that for EBRT patients, *K*^trans^, *k*_ep_, and ADC could successfully distinguish tumor in the PZ. Tumor detection in the CG was hampered by the presence of PU enhancement of *K*^trans^. Recurrent PCa after LDR brachytherapy was challenging to image due to the seeds, causing severe distortions in the DWI, and potentially unreliable ADC values. For these patients, no significant differences were found between ROIs, possibly due to the small sample size.

Using mixed modeling, the mp-MRI combination obtained the best performance in distinguishing tumor and benign tissue on a voxel level. Location significantly contributed to tumor likelihood, mimicking primary tumor prevalence maps in which the PZ has higher tumor incidence. The addition of DCE parameters improved tumor distinction. The decreased accuracy reported by Donati et al [12] may have been caused by the PU enhancement confounder.

The limited prostate coverage by the H&E slides made it impossible to reconstruct the overall shape of the gland, limiting registration accuracy between the slides and MRI. The main study limitation is the small sample size.

In conclusion, when using MRI to guide focal salvage treatments, all imaging modalities should be used for delineation, and the recommended treatment plan should encompass adequate margins beyond the visible tumor to accommodate for size underestimation. Tumor multifocality should also be accounted for. De-escalated whole-gland treatment with a focal tumor boost could potentially fulfill these requisites.

## Electronic supplementary material

ESM 1 (DOCX 894 kb)

## Abbreviations

ADC: Apparent diffusion coefficient
CG: Central gland
DCE-MRI: Dynamic contrast-enhanced MRI
DWI-MRI: Diffusion-weighted imaging MRI
EBRT: External beam radiotherapy
*K*^trans^: Rate of contrast agent transfer from vascular space to extravascular and extracellular space
*k*_ep_: Rate constant from extravascular and extracellular space to vascular space
MRI: Magnetic resonance imaging
mp-MRI: Multi-parametric MRI
PCa: Prostate cancer
PET: Positron emission therapy
PSA: Prostate-specific antigen
PU: Periurethra
PZ: Peripheral zone
RT: Radiation therapy
SRP: Salvage radical prostatectomy
