# Supplementary material for: Quantitative 3-T multi-parametric MRI and step-section pathology of recurrent prostate cancer patients after radiation therapy
Source: Eur Radiol. 2018 Nov 12;29(8):4160–8. doi: 10.1007/s00330-018-5819-y (PMC6610274; doi:10.1007/s00330-018-5819-y)
Supplement: Supplementary file 1 — (DOCX 894 kb) [file 330_2018_5819_MOESM1_ESM.docx]

**Supplementary materials**

**MRI acquisition and post-processing**

The T2w scans were acquired with an echo time (TE) between 90 – 130 ms, and a repetition time (TR) between 2600 – 6000 ms.

The T1w gradient echo scans were acquired with a TE between 1.8 – 2.3 ms and a TR between 3.6 - 5.3 ms. For one patient the T1w sequence was a turbo spin-echo with TE/TR = 8/572 ms.

The DCE sequence was acquired with a TE/TR = 2/4 ms. Contrast injection was followed by a 30 ml saline flush at a flow rate of 3 ml/s.

The apparent diffusion coefficient (ADC) maps were derived from the DWI data excluding b-value = 0. In 12 patients the b-values (s/mm^2^) = 200 and 800 were used; for 2 the used values were b (s/mm^2^) = 500 and 1000 and in 1 patient the values of b (s/mm^2^) = 100 and 1000. For 6 patients, up to 4 b-values between 100 – 800 were used.

Pharmacokinetic analysis was performed with the standard Tofts model [1], using an average T1 value of 1597ms [2] and an arterial input function with parameters derived from an in-house study population.

DCE and DWI- derived maps were generated using MATLAB R2015a (The MathWorks). Further region of interest (ROI) analysis was performed using Python 3 (Python Software Foundation).

[1] Tofts PS, Brix G, Buckley DL, Evelhoch JL, Henderson E, Knopp M V, et al. Contrast-Enhanced T 1 -Weighted MRI of a Diffusable Tracer : Standardized Quantities and Symbols 1999;232:223–32.

[2] de Bazelaire CMJ, Duhamel GD, Rofsky NM, Alsop DC. MR imaging relaxation times of abdominal and pelvic tissues measured in vivo at 3.0 T: preliminary results. Radiology 2004;230:652–9


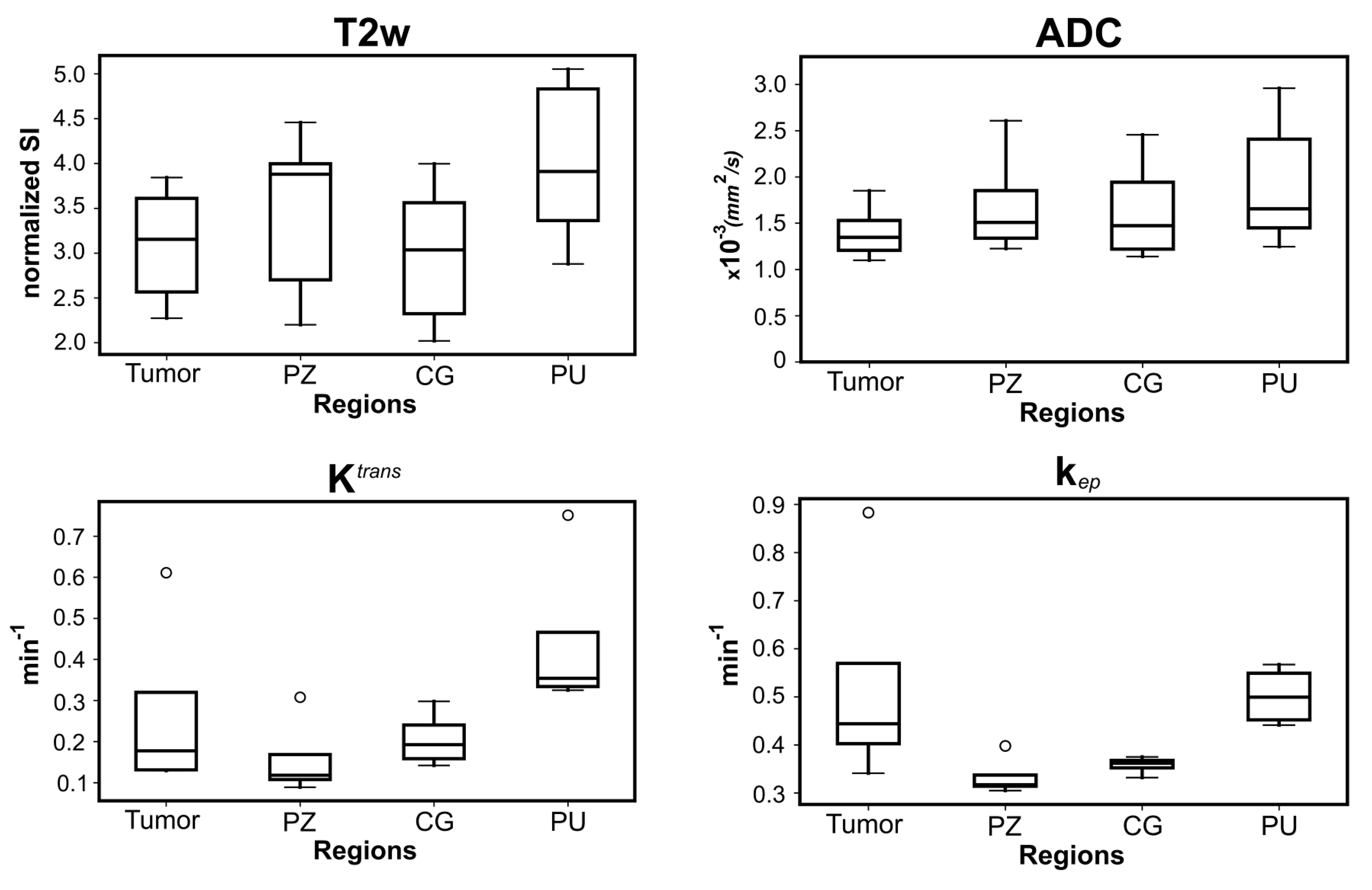


Supplementary Fig. 1. Median imaging values for all ROIs of the six LDR brachytherapy patients. *The boxes represent the first (25^th^) and third (75^th^) quartile; the horizontal line indicates the median and the whiskers the limit Q_1_ – 1.5xQ_1_ and Q_3_ +1.5xQ_3_; dots represent outliers.*


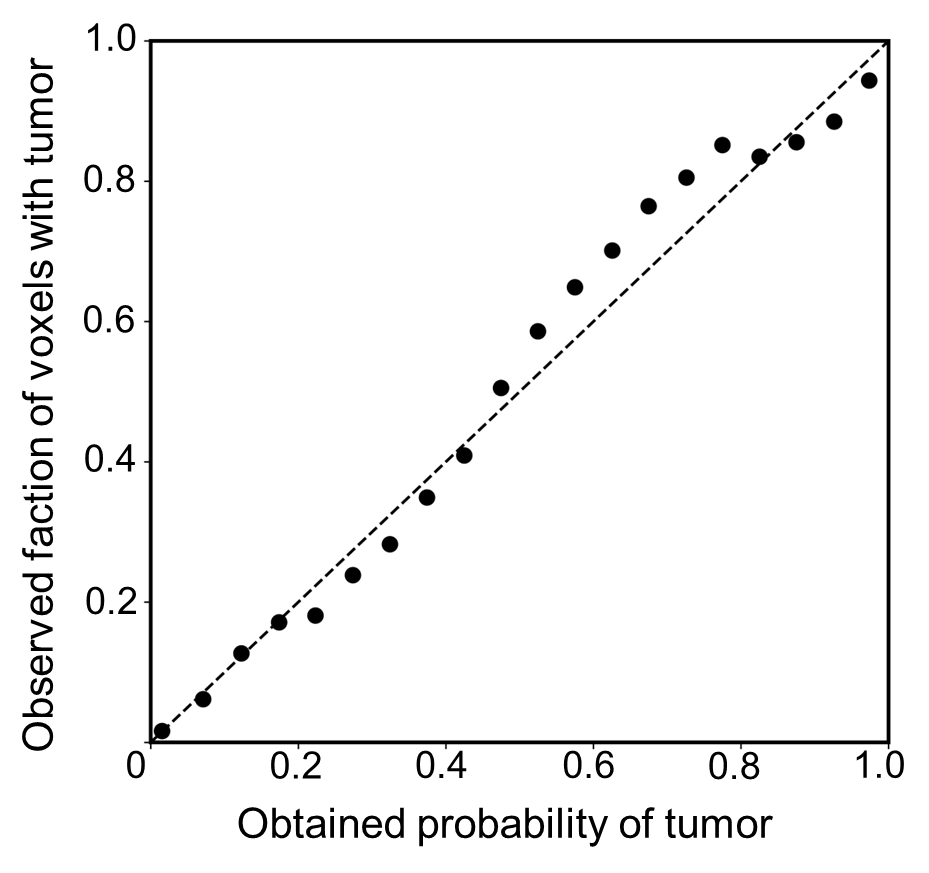


Supplementary Fig. 2. Relation between the fraction of voxels with tumor as observed in histopathology and the probability of tumor as obtained from the model. For voxels within a probability interval of 0.5, the fraction of voxels identified as tumor in the histopathology was calculated. For each interval, this fraction was plotted against the averaged predicted probability by the model. The dashed line represents perfect correspondence between observed fraction and predicted probability.

Supplementary Table 1. Median values for all both subsets of patients and all ROIs. Median (Q10 – Q90).

|  |  |  |  |  |  |  |
| --- | --- | --- | --- | --- | --- | --- |
|  | **EBRT** | | | |  |  |
|  | **Tumor** | **PZ** | **CG** | **PU** |  | |
| T2w (normalized SI) | 2.6 (2.3 - 3.3) | 3.5 (2.3 - 4.2) | 2.8 (2.0 - 3.7) | 3.3 (2.3 - 4.9) |  |  |
| ADC (x10^-3^ mm^2^/s) | 0.9 (0.7 - 1.4) | 1.3 (0.9 - 1.7) | 1.3 (0.9 - 1.5) | 1.4 (1.0 - 1.7) |  |  |
| K^trans^ (min^-1^) | 0.23 (0.15 - 0.33) | 0.13 (0.07 - 0.17) | 0.23 (0.14 - 0.31) | 0.35 (0.23 - 0.45) |  |  |
| k_ep_ (min^-1^) | 0.45 (0.37 - 0.61) | 0.32 (0.24 - 0.37) | 0.35 (0.27 - 0.44) | 0.43 (0.32 - 0.52) |  |  |
|  |  |  |  |  |  |  |
|  | **LDR brachytherapy** | | | |  |  |
|  | **Tumor** | **PZ** | **CG** | **PU** |  |  |
| T2w (normalized SI) | 3.2 (2.4 - 3.7) | 3.9 (2.3 - 4.2) | 3.0 (2.1 - 3.9) | 3.9 (3.1 - 5.0) |  |  |
| ADC (x10^-3^ mm^2^/s) | 1.3 (1.1 - 1.7) | 1.5 (1.3 - 2.3) | 1.5 (1.2 - 2.3) | 1.7 (1.3 - 2.8) |  |  |
| K^trans^ (min^-1^) | 0.18 (0.13 - 0.49) | 0.12 (0.10 - 0.25) | 0.19 (0.15 - 0.27) | 0.35 (0.33 - 0.64) |  |  |
| k_ep_ (min^-1^) | 0.44 (0.37 - 0.76) | 0.32 (0.31 - 0.37) | 0.36 (0.34 - 0.37) | 0.50 (0.45 - 0.56) |  |  |
|  |  |  |  |  |  |  |

Supplementary Table 2. Model parameters obtained when combining T2w, ADC, K^trans^ and k_ep_ imaging values to predict tumor at the voxel-wise level. Imaging values and location are included as fixed effects; patient and spatial coordinates as random effects.

| ***Fixed effects (MRI)*** | Regression coefficients (β) | Std. Error | P |
| --- | --- | --- | --- |
| T2w  ADC reference group (0 – 0.924x10^-3^)  ADC 2^nd^ quartile (0.925x10^-3^ – 1.222x10^-3^)  ADC 3^rd^ quartile (1.223x10^-3^ – 1.438x10^-3^)  ADC 4^th^ quartile (1.439 x10^-3^ – 3.825x10^-3^)  K^trans^ reference group (0 – 0.120)  K^trans^ 2^nd^ quartile (0.121– 0.160)  K^trans^ 3^rd^ quartile (0.161 – 0.248)  K^trans^ 4^th^ quartile (0.249 – 2.494)  k_ep_  Location  Intercept (β_0_) | -0.29  0  -1.70  -2.58  -3.35  0  0.30  1.70  2.26  4.95  -1.44  -2.35 | 0.01  --  0.02  0.02  0.03  --  0.02  0.02  0.03  0.05  0.02  0.41 | <0.001  --  <0.001  <0.001  <0.001  --  0.594  <0.001  <0.001  <0.001  <0.001  <0.001 |
| ***Random effects*** | Variance (σ^2^) |  |  |
| Patients  Distance CM_x_  Distance CM_y_  Distance CM_z_ | 2.43  0.71  0.38  0.35 |  |  |
| ***Residuals (ε_0_)*** | Median | First quartile | Third quartile |
|  | -0.128 | -0.240 | -0.06 |
